# Alveolar Corticotomy-Assisted Orthodontic Closure of a Long-Standing Mandibular First Molar Edentulous Space: A Case Report

**DOI:** 10.7759/cureus.114888

**Published:** 2026-08-20

**Authors:** Keily J Portillo Avila, Richard M Cavero, Ana M Moreno Marin, Yelitza Rojas, Mercedes Dumont

**Affiliations:** 1 Periodontics, Dental Loft, San Pedro Sula, HND; 2 Dentistry, Vedha Family Dentistry, Dover, USA; 3 Dentistry, Signature Dental Group Delray Beach, Delray Beach, USA; 4 Dentistry, Family Dental Home, Casellberry, USA; 5 Dentistry, Family and Cosmetic Dentistry Orlando, Orlando, USA

**Keywords:** accelerated orthodontics, alveolar corticotomy, edentulous space, mandibular first molar, occlusal rehabilitation, orthodontic space closure, orthodontic tooth movement, posterior tooth loss, tooth extraction, young adult

## Abstract

Premature loss of a permanent first molar can lead to functional impairment, food impaction, occlusal instability, and compromised masticatory efficiency. While implant-supported rehabilitation is frequently used to restore missing posterior teeth, orthodontic space closure may provide a conservative alternative in selected patients. This case report describes the management of a 22-year-old healthy female patient who presented with a three-year history of an untreated mandibular right first molar edentulous space following the extraction of tooth #30 at the age of 19. The patient reported recurrent food accumulation within the edentulous area, irritation of the surrounding soft tissues, and difficulty chewing comfortably on the affected side.

A multidisciplinary treatment plan was developed to close the edentulous space through mesial movement of teeth #31 and #32. To facilitate orthodontic tooth movement, alveolar corticotomy was performed under local anesthesia. Multiple cortical perforations were created within the edentulous ridge, followed by placement of a collagen membrane and stabilization with 4-0 nylon sutures. Orthodontic treatment was continued after healing to progressively advance the posterior teeth into the edentulous area.

Substantial space closure was achieved after approximately eight months of active orthodontic movement, followed by additional treatment for alignment and occlusal refinement. Clinical and radiographic evaluation demonstrated favorable healing, successful reduction of the edentulous space, improved posterior occlusal support, elimination of food impaction, and enhanced masticatory function. The patient reported high satisfaction with both the functional and esthetic outcomes.

This case highlights the potential of alveolar corticotomy-assisted orthodontics as a conservative treatment approach for managing long-standing posterior edentulous spaces and demonstrates its ability to facilitate challenging tooth movement while avoiding implant rehabilitation in appropriately selected patients.

## Introduction

Premature loss of a permanent first molar can result in significant functional and occlusal consequences, including drifting of adjacent teeth, food impaction, compromised masticatory efficiency, and altered occlusal relationships. Mandibular first molars are particularly important for maintaining posterior support and overall occlusal stability. When these teeth are lost at a young age and remain unreplaced for extended periods, treatment becomes increasingly challenging because of space reduction, tooth tipping, and alveolar bone remodeling [1-4].

Dental implants are frequently considered a predictable treatment option for replacing missing posterior teeth. However, implant therapy may not always be the most suitable approach for every patient because of anatomical, financial, or treatment-related considerations. In selected cases, orthodontic space closure can provide a conservative alternative by restoring function through controlled movement of adjacent teeth into the edentulous area [5,6].

Alveolar corticotomy is a surgical procedure that involves selective injury to the cortical bone while preserving the surrounding tissues to stimulate localized bone remodeling and facilitate tooth movement. Corticotomy-assisted orthodontics has been shown to accelerate orthodontic tooth movement and improve the predictability of complex space closure procedures, particularly in patients with long-standing edentulous areas [7-10]. In addition, randomized controlled trials have demonstrated that alveolar corticotomy can accelerate tooth movement while maintaining dentoalveolar stability [11]. Orthodontic tooth movement is mediated by complex biological and molecular mechanisms involving coordinated bone resorption and formation in response to mechanical forces. Corticotomy-assisted approaches may further enhance these biological responses through increased regional bone remodeling and osteocyte-mediated mechanotransduction [12-14].

Cone-beam computed tomography (CBCT) can provide three-dimensional assessment of the alveolar ridge, tooth positions, available bone volume, and surrounding anatomical structures when such information is required for orthodontic treatment planning [15,16]. In the present case, CBCT was obtained after placement of fixed orthodontic appliances and used to evaluate the long-standing edentulous ridge, adjacent posterior teeth, available bone volume, and surrounding anatomical structures to support planning for corticotomy-assisted orthodontic space closure. CBCT was used as a diagnostic and treatment-planning aid rather than as a quantitative method for evaluating treatment outcomes.

This report describes the management of a 22-year-old female patient with a three-year history of an untreated mandibular first molar edentulous space following extraction of tooth #30. A multidisciplinary treatment approach combining alveolar corticotomy and orthodontic space closure was used to facilitate mesial movement of the mandibular posterior teeth, improve posterior occlusal support, and avoid implant placement.

## Case presentation

A 22-year-old female patient (ASA I) with no known drug allergies presented for evaluation of a long-standing edentulous space in the mandibular right posterior region. The patient reported that tooth #30 had been extracted at 19 years of age due to extensive dental destruction. Following extraction, the edentulous space remained untreated for approximately three years. During this period, the patient experienced recurrent food impaction within the area, irritation of the adjacent soft tissues, and difficulty chewing comfortably on the affected side.

**Figure 1:**
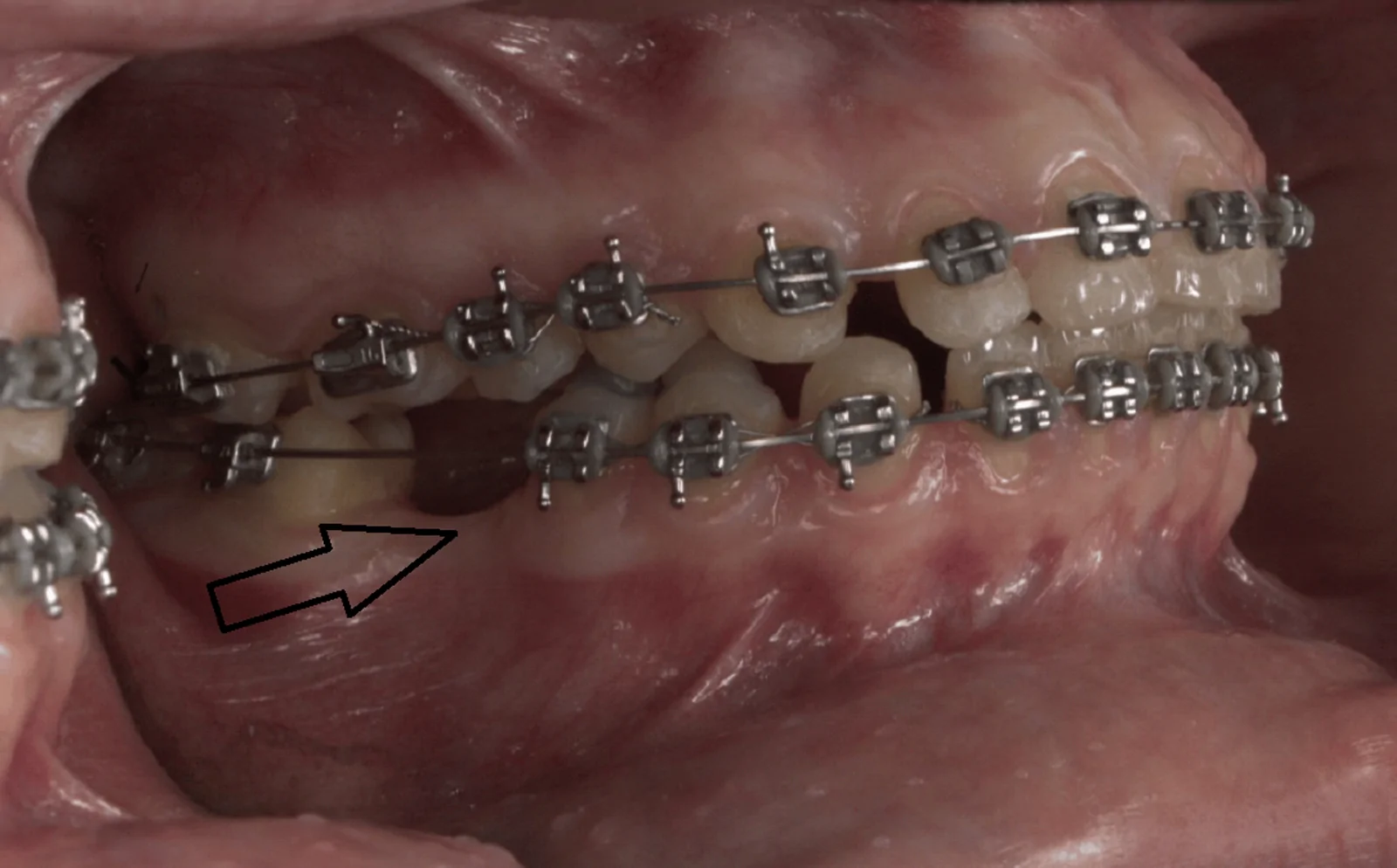
Initial Clinical Presentation of the Long-Standing Edentulous Space. Initial clinical right lateral intraoral view demonstrating the long-standing edentulous space in the mandibular right posterior region. Fixed orthodontic appliances were present as part of the initial orthodontic treatment phase. The arrow indicates the edentulous space corresponding to the previously extracted tooth #30, which had remained untreated for approximately three years before corticotomy-assisted orthodontic space closure.

Clinical examination revealed a persistent edentulous space corresponding to tooth #30. Clinical and radiographic evaluation demonstrated reduced posterior occlusal support and findings consistent with mesial migration of the adjacent teeth (Figure 1).

**Figure 2:**
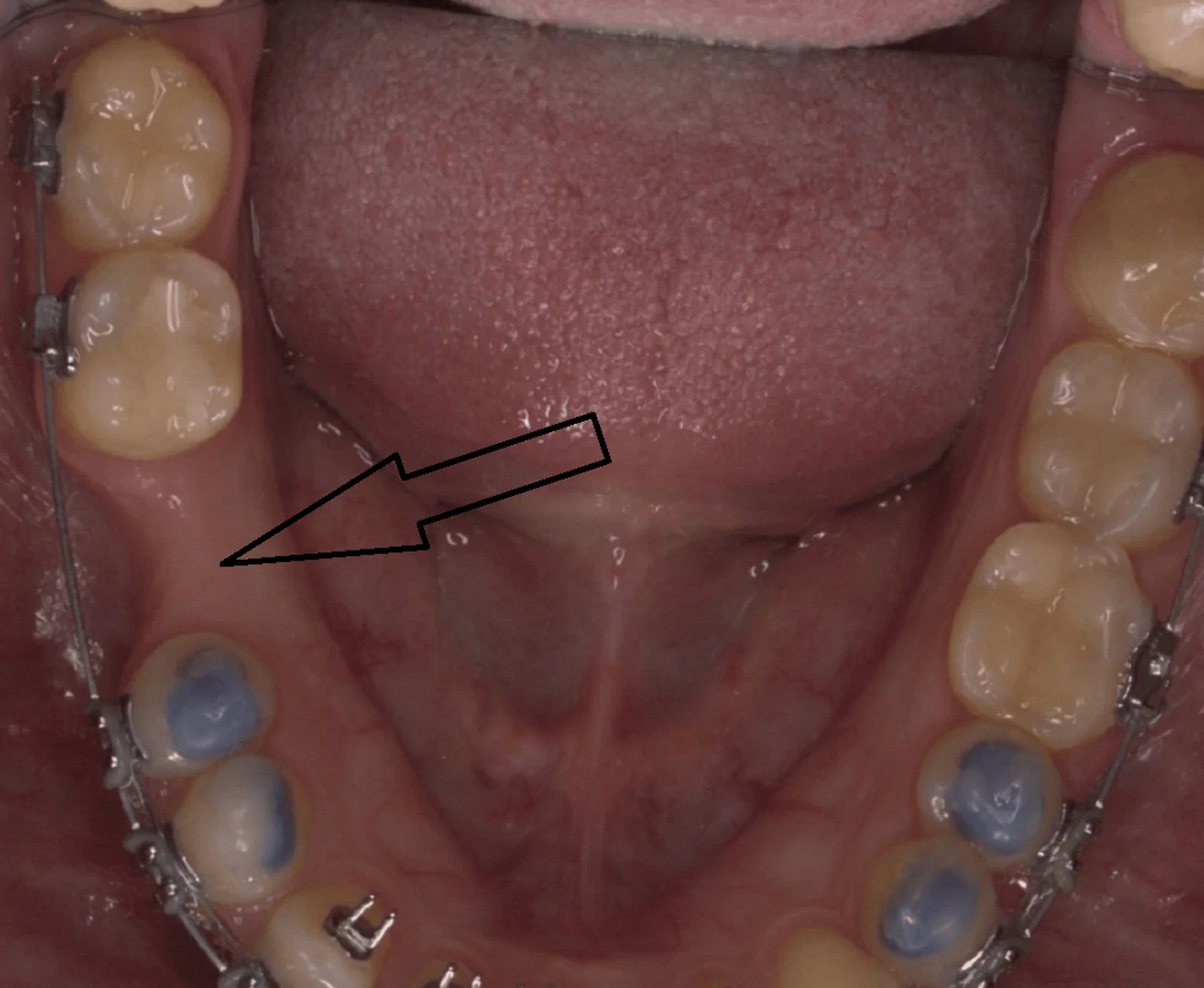
Occlusal View of the Long-Standing Edentulous Space Prior to Corticotomy. Occlusal view of the mandibular arch demonstrating the long-standing edentulous space corresponding to the previously extracted tooth #30. Fixed orthodontic appliances were in place as part of the initial alignment phase and preparation for orthodontic space closure. The arrow indicates the edentulous ridge that was selected for corticotomy-assisted orthodontic tooth movement and subsequent mesialization of the mandibular posterior teeth.

An occlusal view of the mandibular arch further demonstrated the extent of the edentulous area and the presence of fixed orthodontic appliances that had been placed to initiate alignment and establish favorable biomechanics for future space closure (Figure 2).

**Figure 3:**
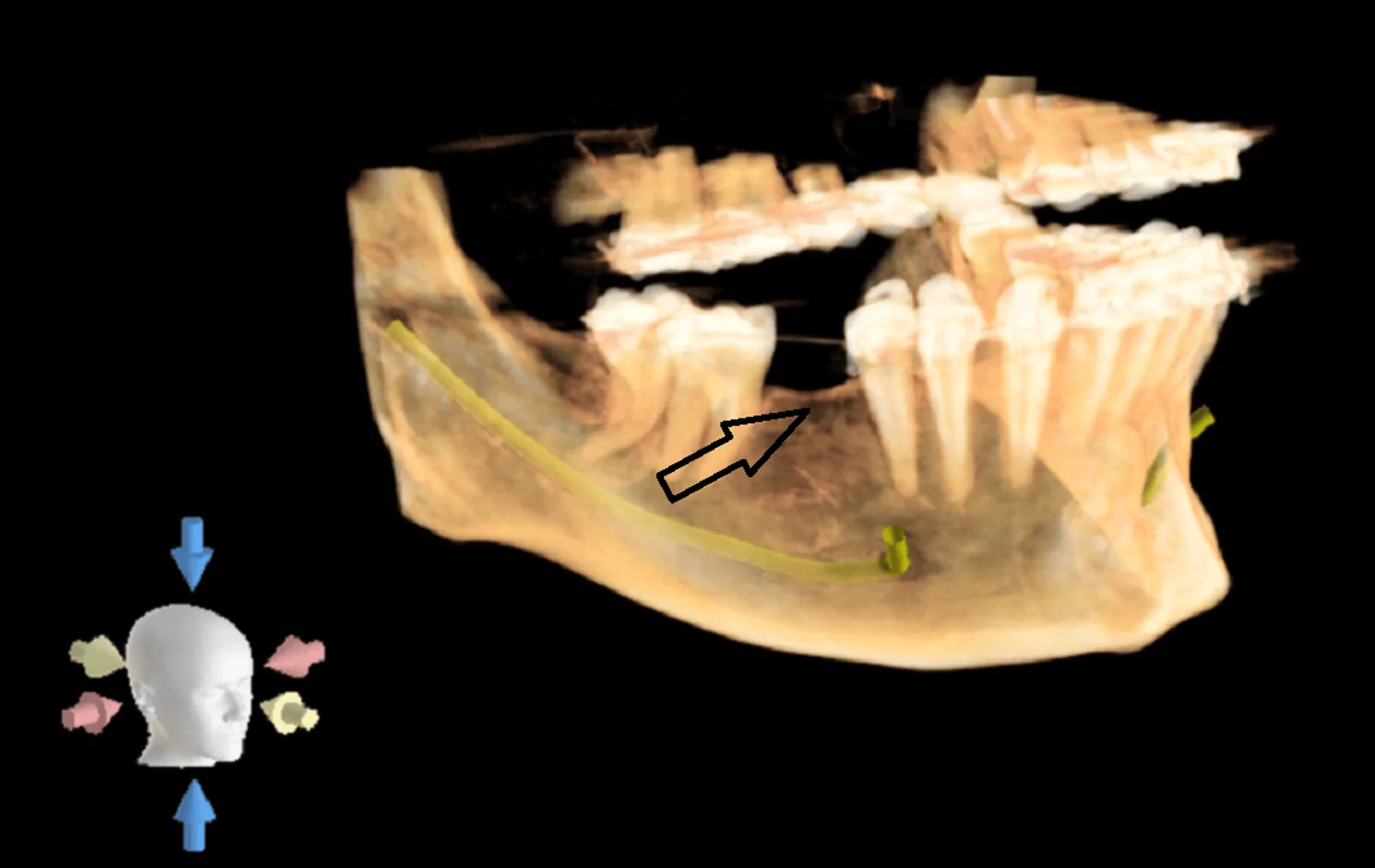
Three-Dimensional CBCT Evaluation for Corticotomy-Assisted Orthodontic Space Closure. Three-dimensional cone-beam computed tomography (CBCT) reconstruction obtained after placement of the fixed orthodontic appliances during the orthodontic treatment-planning phase. The complete CBCT examination was used to evaluate alveolar ridge morphology, adjacent tooth positions, available bone volume, and surrounding anatomical structures to plan corticotomy-assisted orthodontic space closure. The three-dimensional reconstruction shown is presented as a representative illustration of the case.

Three-dimensional CBCT was obtained after placement of the fixed orthodontic appliances as part of the orthodontic treatment-planning phase for corticotomy-assisted space closure. The complete CBCT examination was used to evaluate the alveolar ridge morphology, adjacent tooth positions, available bone volume, and surrounding anatomical structures. The three-dimensional reconstruction shown in Figure 3 is a representative view from this examination and was used as a diagnostic and treatment-planning aid rather than a quantitative method for evaluating treatment outcomes.

Following a comprehensive orthodontic assessment, a multidisciplinary treatment plan was developed to achieve closure of the edentulous space through mesial movement of teeth #31 and #32 as an alternative to implant rehabilitation. Orthodontic treatment was initiated to establish proper alignment and create favorable biomechanics for posterior tooth movement. To facilitate mesialization of the mandibular posterior teeth and reduce resistance within the cortical bone, alveolar corticotomy was planned in the edentulous area. Previous studies have reported that this surgical approach may reduce overall treatment duration while demonstrating minimal adverse effects on root structure during complex tooth translation.

**Figure 4:**
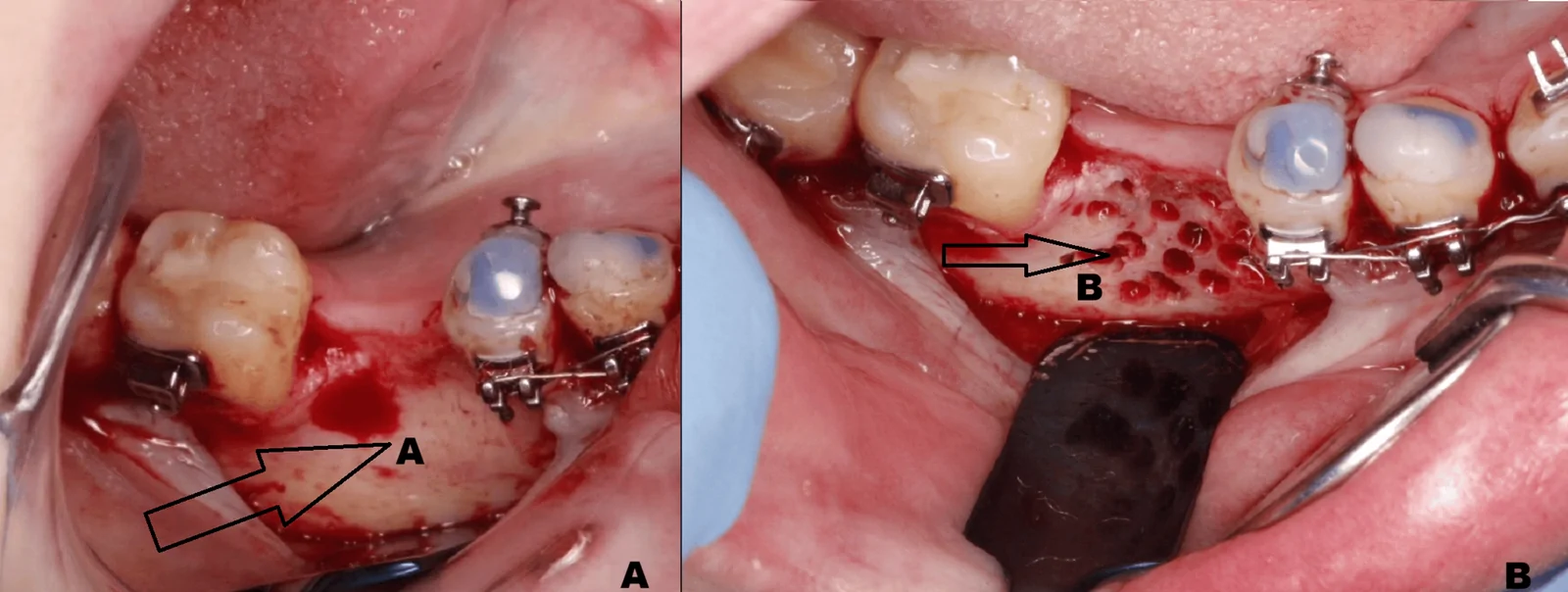
Alveolar Corticotomy Procedure Performed Within the Edentulous Space. (A) Following flap elevation, the cortical bone within the edentulous area corresponding to the previously extracted tooth #30 was exposed. The arrow indicates the alveolar ridge selected for corticotomy-assisted orthodontic tooth movement. (B) Multiple cortical perforations were created using a medium round bur mounted on a surgical handpiece. The arrow indicates the corticotomy perforations performed to stimulate localized bone remodeling and facilitate mesial movement of the mandibular posterior teeth during orthodontic space closure.

Under local anesthesia using 2% lidocaine with 1:100,000 epinephrine, a full-thickness flap was elevated over the edentulous ridge. The cortical bone within the edentulous area was exposed, and multiple cortical perforations were created using a medium round bur mounted on a surgical handpiece (Figure 4).

The perforations were distributed throughout the cortical bone to stimulate localized bone remodeling and facilitate orthodontic tooth movement. Systematic reviews have demonstrated that periodontally accelerated osteogenic orthodontics enhances tooth movement velocity while supporting alveolar bone thickness throughout treatment.

**Figure 5:**
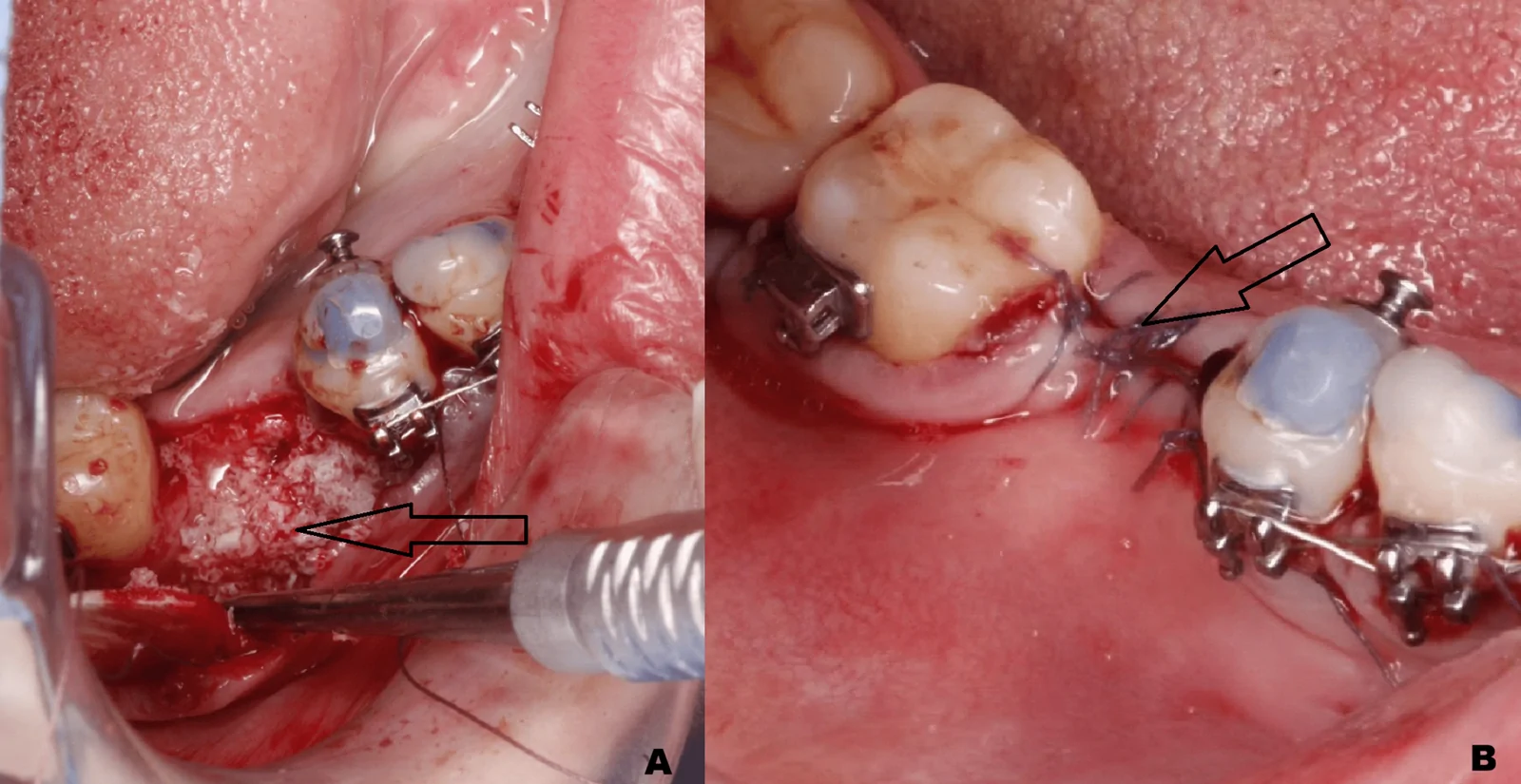
Collagen Membrane Placement and Surgical Closure Following Alveolar Corticotomy. (A) Placement of a collagen membrane over the corticotomy site following completion of the cortical perforations. The membrane was used to cover the treated area and support soft tissue healing and stabilization. (B) Repositioning and suturing of the surgical flaps using 4-0 nylon sutures to achieve primary closure of the surgical site. Sutures were maintained for 15 days before removal, resulting in favorable postoperative healing.

Following completion of the corticotomy, a collagen membrane was placed over the treated area to protect the surgical site and support tissue stabilization. The surgical flaps were subsequently repositioned and secured using 4-0 nylon sutures to achieve primary closure (Figure 5).

Postoperative healing progressed uneventfully, and the sutures were removed after 15 days. No complications, such as infection, wound dehiscence, or excessive postoperative discomfort, were observed.

**Figure 6:**
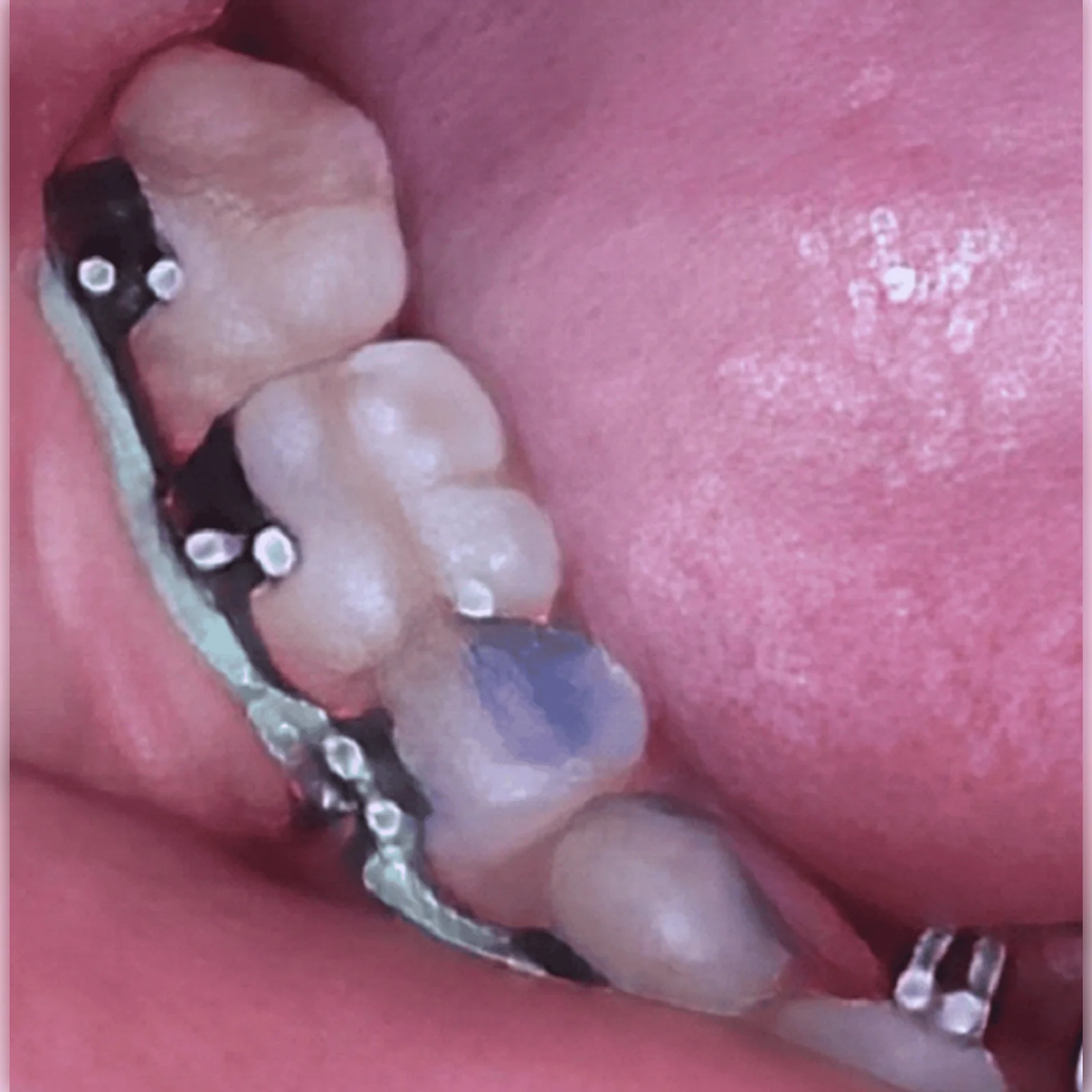
Clinical Progress Following Corticotomy-Assisted Orthodontic Tooth Movement. Follow-up intraoral photograph demonstrating the clinical progress of corticotomy-assisted orthodontic movement of the mandibular right posterior teeth toward the edentulous region corresponding to the previously extracted tooth #30. Substantial reduction of the edentulous space was observed following approximately eight months of active orthodontic movement. Fixed orthodontic appliances remained in place because treatment was ongoing for further space closure, alignment, and occlusal refinement.

Orthodontic mechanics were resumed following healing, allowing progressive mesial movement of teeth #31 and #32 into the edentulous space. A substantial reduction of the edentulous area was observed over approximately eight months of active orthodontic movement. Follow-up clinical evaluation demonstrated favorable soft tissue healing, continued tooth movement, and substantial reduction of the edentulous space, demonstrating favorable clinical progress during the ongoing corticotomy-assisted orthodontic treatment (Figure 6).

At the time of this report, orthodontic treatment remains ongoing to achieve complete space closure, final alignment, and occlusal refinement. The patient reported improvement in chewing efficiency, reduced food impaction, and satisfaction with the functional and esthetic progress achieved thus far.

## Discussion

Premature loss of a mandibular first molar can lead to a variety of functional and occlusal complications, including food impaction, drifting of adjacent teeth, reduced posterior support, and decreased masticatory efficiency. These consequences may become more pronounced when the edentulous space remains untreated for an extended period, as alveolar remodeling and tooth movement can complicate future rehabilitation [1,2]. In the present case, the patient presented with a three-year history of an untreated edentulous space following extraction of tooth #30, resulting in functional discomfort and difficulty chewing.

Dental implants are frequently considered a treatment option for replacing missing posterior teeth because they can restore function without affecting adjacent dentition. However, implant therapy may not be the ideal solution in every clinical situation. Patient age, treatment objectives, financial considerations, available bone volume, and long-term maintenance requirements may influence treatment planning. In carefully selected patients, orthodontic space closure can provide a conservative alternative by restoring function through movement of the natural dentition rather than prosthetic replacement [3,4].

Alveolar corticotomy has been used as an adjunctive procedure to facilitate orthodontic tooth movement by stimulating localized bone remodeling [5-7]. The biologic response associated with corticotomy may reduce resistance within the cortical bone and enhance the rate of tooth movement [8-13]. This approach may be particularly useful in cases involving long-standing edentulous spaces, where conventional orthodontic movement may require extended treatment times [14-16].

In the present case, corticotomy-assisted orthodontics was selected to facilitate mesial movement of teeth #31 and #32 into the edentulous area. Clinical follow-up demonstrated substantial reduction of the edentulous space, favorable healing of the surgical site, and progressive movement of the posterior teeth. No postoperative complications were observed, and the patient reported improvement in chewing function and reduction of food impaction. These findings suggest that corticotomy-assisted orthodontics may serve as a useful adjunct when significant tooth movement is required within previously edentulous regions.

An additional potential advantage of this treatment approach is the preservation of natural dentition and avoidance of implant placement. In this case, movement of the patient's existing teeth provided progressive improvement in arch continuity without prosthetic replacement. Although orthodontic treatment remains ongoing, the clinical progress observed thus far suggests potential benefits of combining corticotomy with orthodontic mechanics for the management of selected posterior edentulous spaces.

This case highlights the importance of interdisciplinary treatment planning and suggests that corticotomy-assisted orthodontic space closure may represent a conservative treatment option for carefully selected patients presenting with long-standing mandibular first molar edentulous spaces.

## Conclusions

In this case, alveolar corticotomy-assisted orthodontics was associated with favorable clinical progress in the management of a long-standing mandibular first molar edentulous space. The procedure was associated with progressive mesial movement of the mandibular posterior teeth, substantial reduction of the edentulous area, and improvement in functional arch continuity without implant rehabilitation. Favorable healing and continued orthodontic tooth movement were observed, while the patient reported improvement in masticatory function. Although treatment remains ongoing, this case suggests that corticotomy-assisted orthodontic space closure may represent a conservative treatment option for carefully selected patients with posterior edentulous spaces and highlights the value of interdisciplinary collaboration between orthodontic and surgical approaches.
